# Shivering During Transurethral Ureterolithotripsy Predicted Postoperative Sepsis: A Case Report

**DOI:** 10.7759/cureus.97924

**Published:** 2025-11-27

**Authors:** Yuko Kato (Araki), Gaku Kawamura, Michiko Ushio, Masahiko Bougaki, Kanji Uchida

**Affiliations:** 1 Anesthesiology and Pain Relief Center, The University of Tokyo Hospital, Tokyo, JPN; 2 Anesthesiology, Institute of Medical Science, University of Tokyo, Tokyo, JPN

**Keywords:** anesthesia, chills, sepsis, transurethral lithotripsy, tremors

## Abstract

Patients may develop septic shock following transurethral ureterolithotripsy (TUL). However, intraoperative symptoms that may be indicative of sepsis remain largely understudied. We encountered a case in which shivering during spinal anesthesia predicted bacteremia, consequently enabling early therapeutic intervention for sepsis. A 68-year-old woman had diabetes, severe obesity (BMI 45 kg/m²), sleep apnea syndrome, hypertension, and dyslipidemia. The patient was hospitalized multiple times for calculous pyelonephritis due to a right, coral-shaped calculus and received antimicrobial therapy. TUL was scheduled for the right coral-shaped calculus. Spinal anesthesia was administered, and surgery was initiated after confirming loss of cold sensation at T4. The patient developed severe shivering, nausea, and vomiting approximately 50 minutes after lithotripsy initiation. Vital signs showed no significant changes compared with those at admission and did not indicate shock. However, the symptoms were considered initial signs of bacteremia. Therefore, surgery was terminated. Arterial and central venous lines were inserted while the patient was in the operating room, before admission to the ICU. Upon ICU admission, the patient presented with hypotension (systolic blood pressure ≤70 mmHg) and hyperlactatemia. The patient was consequently diagnosed with septic shock. Norepinephrine, dobutamine, and respiratory support were initiated with noninvasive positive pressure ventilation. Tracheal intubation and vasopressin administration were performed the day after surgery. Thermoregulatory shivering can occur during spinal anesthesia, owing to the use of intrapyelic perfusion fluid during lithotripsy. However, the shivering observed in this case was highly likely to be the initial symptom of bacteremia. Furthermore, the patient had a preoperative urinary tract infection, suggesting a high risk for sepsis and endotoxic shock. This case confirms that chills and rigors may be early indicators of bacteremia. Moreover, it suggests that intraoperative shivering is an initial symptom of bacteremia.

## Introduction

Transurethral ureterolithotripsy (TUL) is an endoscopic surgery for treating ureteral stones. A thin endoscope is inserted through the urethra to directly view the stone inside the ureter, and the stone is broken up into small pieces with a laser and then removed. Sepsis is recognized as a complication of TUL. Serious complications may occur after TUL. In a Japanese study, 296 (2.39%) out of 12,372 patients undergoing TUL developed serious complications [1]. Cases requiring catecholamine administration due to septic shock or similar conditions accounted for 1.94% of all cases [1]. For septic shock, comprehensive protocols and early goal-directed therapy have shown both reductions in mortality and improved resource utilization, supporting guideline recommendations for intervention within the first few hours of diagnosis. [2,3]. Therefore, early detection of sepsis remains critical even in patients undergoing surgical anesthesia management. However, few reports have described intraoperative signs as diagnostic triggers [4]. Shivering during spinal anesthesia is usually benign because it reflects a harmless thermoregulatory response, not a sign of instability or serious disease. Herein, we report a case in which shivering during spinal anesthesia predicted sepsis in a woman in her 60s undergoing TUL for calculous pyelonephritis, thereby enabling early therapeutic intervention.

This article was previously presented as a meeting abstract at the 2017 Japanese Society of Intensive Care Medicine Annual Scientific Meeting on March 10, 2017.

## Case presentation

A 68-year-old woman (height: 140 cm, weight: 88.4 kg, BMI: 45.1 kg/m²) was admitted to our hospital for calculous pyelonephritis. The patient had a history of sleep apnea syndrome, diabetes, hypertension, and dyslipidemia. She had recurrent right ureteric staghorn calculi and associated pyelonephritis, for which TUL was considered. However, conservative management was adopted because of the patient’s multiple comorbidities, high risk of complications, and preserved renal function. The patient experienced pyelonephritis recurrence four months before surgery. A right ureteral stent was placed, and further conservative treatment was deemed difficult. Thus, TUL was scheduled for the right ureteral staghorn calculus.

**Figure 1:**
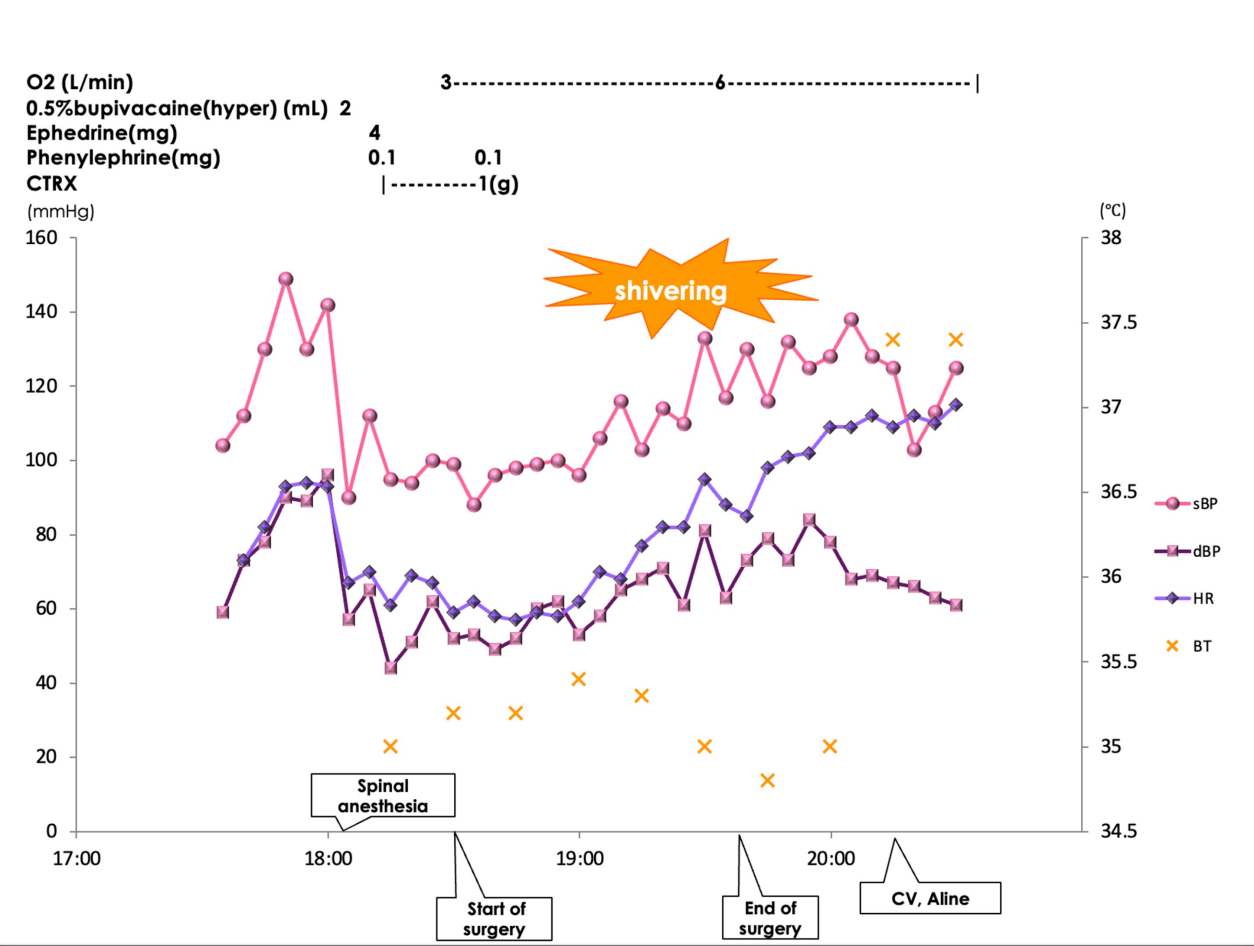
Intraoperative vital signs and administration of major drugs The course of the surgery is shown in the figure. Approximately 50 minutes after the start of surgery, the patient exhibited shivering and vomiting. The surgery was halted, and an arterial line was inserted into the radial artery, while a central venous line was inserted into the right jugular vein. sBP: systolic blood pressure; dBP: diastolic blood pressure; HR: heart rate; BT: body temperature; CTRX: ceftriaxone sodium hydrate; CV: central venous line, A-line: arterial line

Spinal anesthesia was selected as the anesthetic method. Upon admission to the operating room, the blood pressure, pulse rate, respiratory rate, and SpO2 were 113/73 mmHg, 77 beats/minute, 23/minute, and 95% (room air), respectively. A 0.5% hyperbaric bupivacaine solution (10 mg) was administered at the L3/4 interspace, and loss of cold sensation was confirmed at T4. One gram of ceftriaxone sodium hydrate was administered before surgery. The patient complained of right flank pain approximately 40 minutes after lithotripsy began. Shivering occurred approximately 50 minutes after lithotripsy, followed by nausea and vomiting. Vital signs showed no significant changes compared to those at admission and did not indicate shock. However, the anesthesiologist considered that shivering during lithotripsy of an infected stone was highly suggestive of early sepsis. After consulting the surgeon and conveying these concerns, the procedure was terminated. Arterial and central venous lines were inserted into the patient while in the operating room, followed by transfer to the ICU (Figure 1).

Upon ICU admission, the patient’s temperature, pulse rate, blood pressure, respiratory rate, and SpO_2_ were 38.8°C, 126 beats/minute, 112/64 mmHg, 25/min, and 99% (on 8 L/minute oxygen), respectively. Although fluid loading continued from the operating room, a drop in systolic blood pressure to <70 mmHg was observed immediately after ICU admission. The patient required circulatory support and was administered norepinephrine and dobutamine. Respiratory support was provided using noninvasive positive pressure ventilation. Blood tests the following day revealed elevated procalcitonin (156.45 ng/mL), high levels of inflammatory markers (white blood cells: 30,900/μL, C-reactive protein: 12.19 mg/dL), and hyperlactatemia (5.5 mmol/L), leading to a diagnosis of septic shock (Table 1).

**Table 1:** Perioperative blood test trends WBC: white blood cells; CRP: C-reactive protein; PCT: procalcitonin

Test	Normal range	Before the surgery	Immediately after the surgery	The day after the surgery
WBC (/μL)	3,500 - 9,200	4,600	23,400	30,900
CRP (mg/dL)	0.00 - 0.30	0.49	4.94	12.19
Lactate (mmol/L)	0.5 - 1.5	Not available	2.7	5.5
PCT (ng/mL)	0 - 0.49	Not available	Not available	156.45

**Figure 2:**
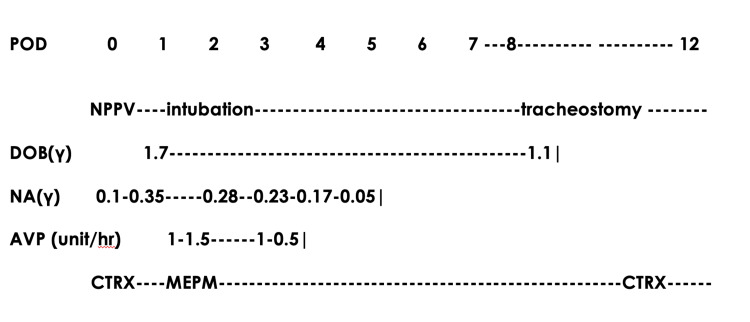
Postoperative ICU progress and administration of major drugs The patient received circulatory support with vasopressors and was intubated on POD 1. Vasopressors were gradually tapered and discontinued on POD 8. A tracheotomy was performed on POD 8, mechanical ventilation was terminated on POD 11, and the patient was discharged from the ICU on POD 13. POD: postoperative days; NPPV: noninvasive positive pressure ventilation; DOB: dobutamine; NA: norepinephrine; AVP: vasopressin; CTRX: ceftriaxone sodium hydrate; MEPM: meropenem; γ: micrograms/kilograms/minute.

The patient was intubated and placed on mechanical ventilation on postoperative day 1. Vasopressin was administered because of hypotension. As the extubation criteria were not met, tracheostomy was performed on postoperative day 8. The patient was weaned off the ventilator on postoperative day 11 and discharged from the ICU on postoperative day 13 (Figure 2). The patient was discharged home on the 68^th^ postoperative day.

## Discussion

This case demonstrates that septic shock can occur after TUL and that shivering during spinal anesthesia may be an initial symptom of sepsis.

Patients presenting with chills and rigors in the emergency department have a higher likelihood of bacteremia [5,6]. This case demonstrated postoperative procalcitonin elevation and septic shock requiring massive catecholamine administration. To the best of our knowledge, no previous study has indicated that symptoms during TUL can predict postoperative sepsis.

It is difficult to distinguish between shivering as an early symptom of sepsis and harmless shivering during spinal anesthesia. Shivering is not specific to sepsis. Non-thermoregulatory shivering may occur alongside thermoregulatory shivering during spinal anesthesia [7]. During TUL, the retrograde flow of endotoxins or bacterial clusters into the systemic circulation, accompanied by an increase in intrapyelic pressure, may induce septic shock. Therefore, it is crucial that anesthesiologists share surgical procedures with urologists. In particular, the use of retrograde ureteral irrigation fluid during lithotripsy is highly likely to induce sepsis, and the shivering observed at this time requires special attention.

Coral-shaped stones extend from the renal calyx to the renal pelvis. They often present treatment challenges owing to their large volume and frequent association with infections [8]. In our case, the patient had a history of recurrent urinary tract infections before surgery, which suggested a high risk of sepsis. Urological surgical procedures are associated with an increased risk of bacterial and endotoxin entry into the bloodstream due to elevated renal pelvic pressure from irrigation fluids [9]. Patient death due to sepsis following TUL has also been reported [10]. Although the management in the present case could be considered over-triage, early initiation of sepsis treatment is essential.

Line insertion was difficult in this patient because of severe obesity complicated by diabetes. Given the late time, early diagnosis before ICU admission and initiation of treatment in the operating room were considered significant.

## Conclusions

Sepsis-induced shock can occur after TUL. Shivering that occurs during the use of intrapyelocalyceal irrigation fluid during lithotripsy may be an initial symptom of sepsis, necessitating careful observation of the patient's systemic condition during surgical anesthesia management. This is a single case report, and it is difficult to distinguish between benign shivering and shivering as a warning sign of sepsis. We believe that additional cases should be accumulated and examined to determine whether intraoperative shivering is a useful predictor of sepsis in patients after TUL.

## References

[1] Sugihara T, Yasunaga H, Horiguchi H (2013). A nomogram predicting severe adverse events after ureteroscopic lithotripsy: 12  372 patients in a Japanese national series. BJU Int.

[2] Rivers EP, McIntyre L, Morro DC, Rivers KK (2005). Early and innovative interventions for severe sepsis and septic shock: taking advantage of a window of opportunity. CMAJ.

[3] De Waele JJ (2024). Importance of timely and adequate source control in sepsis and septic shock. J Intensive Med.

[4] Kim HJ, Choi YS, Jin JH, Lee B (2022). Management of pulmonary aspiration due to undiagnosed achalasia during induction of general anesthesia - a case report. Anesth Pain Med (Seoul).

[5] Tokuda Y, Miyasato H, Stein GH, Kishaba T (2005). The degree of chills for risk of bacteremia in acute febrile illness. Am J Med.

[6] Lee CC, Wu CJ, Chi CH (2012). Prediction of community-onset bacteremia among febrile adults visiting an emergency department: rigor matters. Diagn Microbiol Infect Dis.

[7] Crowley LJ, Buggy DJ (2008). Shivering and neuraxial anesthesia. Reg Anesth Pain Med.

[8] Codău CA, Coman F, Novac B (2024). Challenging management of coralliform kidney and ureteral stones. Arch Clin Cases.

[9] Troxel SA, Low RK (2002). Renal intrapelvic pressure during percutaneous nephrolithotomy and its correlation with the development of postoperative fever. J Urol.

[10] Dassanayake SN, Harrison NL, Traxer O, Gauhar V, Yuen SK, Somani BK (2025). Mortality from ureteroscopy for kidney stone disease: systematic review of literature from the section of EAU endourology. World J Urol.

